# Hybrid artificial intelligence framework for patient emotion recognition in dentistry

**DOI:** 10.3389/froh.2026.1909407

**Published:** 2026-07-29

**Authors:** Aryadi Aryadi, Harris Gadih Pratomo, Kanoksak Wattanachote, Taechinee Ratanawimon, Phawit Phanchantraurai, Wanlida Suphasri-itsara, Panupat Phumpatrakom, Samroeng Inglam, Siriwan Suebnukarn

**Affiliations:** 1 Faculty of Dentistry, Trisakti University, Jakarta, Indonesia; 2 Faculty of Information and Communication Technology, Mahidol University, Nakorn Pathom, Thailand; 3 Faculty of Dentistry, Thammasat University, Pathum Thani, Thailand

**Keywords:** artificial intelligence, dental consultation, emotion analysis, large language model, patient emotion recognition

## Abstract

**Introduction:**

Emotional communication plays an important role in dentist-patient interactions, yet domain-specific resources for automated emotion recognition in dental consultations remain limited.

**Objective:**

This study aimed to develop an emotional dental consultation corpus derived from restorative dentistry consultations and to evaluate a hybrid artificial intelligence (AI) framework for automated patient emotion recognition.

**Methods:**

Audio recordings from 12 restorative dentistry consultations were transcribed and segmented into patient utterances. A total of 2,160 utterances were independently annotated into six emotion categories (Neutral, Fear, Sad, Happy, Angry, and Disgust) by three trained annotators using a standardized annotation framework. Inter-annotator reliability was assessed using Cohen's kappa coefficient. The resulting corpus was used to train a Bidirectional Long Short-Term Memory (BiLSTM) classifier and transformer-based baseline models, including BERT and RoBERTa. A hybrid framework was developed by applying large language model (LLM)-based confidence re-ranking to low-confidence BiLSTM predictions. Three open-source LLMs (Llama 3.1, DeepSeek-R1, and Qwen 2.5) were evaluated.

**Results:**

The developed corpus demonstrated high annotation reliability (*κ* = 0.89). Neutral (23.15%), Fear (19.44%), and Disgust (18.98%) were the most prevalent emotion categories. Fear was both one of the most frequently occurring emotions and the most challenging category to classify. The baseline BiLSTM model achieved a macro-F1 score of 0.87, whereas BERT and RoBERTa achieved macro-F1 scores of 0.89 and 0.91, respectively. The hybrid frameworks achieved numerically higher performance, with the BiLSTM + Qwen 2.5 framework obtaining the highest macro-F1 score of 0.92. However, the observed performance improvements were not statistically significant (all *p* > 0.05).

**Conclusion:**

This study presents a novel emotion-annotated dental consultation corpus and demonstrates the feasibility of a confidence-based hybrid AI framework for automated patient emotion recognition in dentistry. Although the proposed framework achieved the highest numerical performance, the findings should be regarded as preliminary evidence of the potential utility of selective LLM-based re-ranking for recognizing emotionally ambiguous patient utterances.

## 1 Introduction

Effective communication between dentists and patients is a fundamental component of patient-centered dental care. Beyond the exchange of clinical information, dental consultations involve complex emotional interactions that can influence patient trust, treatment acceptance, adherence to recommendations, and overall satisfaction with care. Patients frequently experience emotions such as fear, anxiety, uncertainty, frustration, or relief during discussions of diagnosis, treatment options, and expected outcomes. These emotional responses are particularly prevalent in dentistry, where concerns regarding pain, treatment complexity, financial costs, and procedural outcomes may affect patients' perceptions and decision-making. Studies have consistently shown that dental anxiety is common and can negatively influence dental attendance, treatment acceptance, and oral health outcomes (1, 2). Furthermore, anxiety and pain-related concerns are closely linked to patients' experiences during dental treatment (3). Consequently, the ability to recognize and respond appropriately to patient emotions is an essential communication skill for dental professionals and plays a critical role in establishing effective therapeutic relationships.

Recent advances in artificial intelligence (AI) and natural language processing (NLP) have enabled the development of automated emotion recognition systems capable of identifying emotional states from spoken or written language. Emotion recognition has become an important area of affective computing (4), with applications in healthcare, mental health assessment, telemedicine, customer service, and human-computer interaction. Affect detection has attracted increasing interdisciplinary interest because of its potential to improve human-centered technologies and support adaptive interactions (5). Deep learning approaches, including recurrent neural networks and transformer-based architectures, have demonstrated promising performance in detecting emotions from textual data, while advances in multimodal affective computing have further enhanced recognition capabilities through the integration of linguistic, acoustic, and visual information (6). In healthcare settings, automated emotion recognition has the potential to support communication analysis, patient monitoring, and decision-support systems by providing objective assessments of patients' emotional states during clinical interactions.

Despite these advances, the development of reliable emotion recognition systems remains heavily dependent on the availability of high-quality annotated corpora. Existing emotion datasets are predominantly derived from social media posts, movie dialogues, customer service interactions, or general conversational datasets. Many of these corpora are based on established emotion frameworks, including Ekman's theory of basic emotions (7). Widely used benchmark datasets include IEMOCAP (8), EmotionLines (9), and MELD (10), which have played important roles in advancing emotion recognition research. Although several healthcare-related corpora have been developed, few are specifically designed to capture the unique emotional characteristics of dental consultations. Dental encounters differ substantially from other healthcare interactions because they frequently involve emotions associated with dental anxiety, fear of pain, uncertainty regarding treatment outcomes, embarrassment about oral conditions, and relief following diagnosis or treatment planning. As a result, emotion recognition models trained on general-domain datasets may not adequately capture the linguistic and contextual nuances of patient emotional expressions in dental settings. To date, there remains a lack of rigorously annotated emotion corpora derived from authentic dentist-patient consultations, limiting the development and evaluation of domain-specific emotion recognition systems for dentistry.

The application of AI to healthcare communication has expanded rapidly in recent years. Machine learning approaches have demonstrated considerable potential for supporting clinical decision-making, patient monitoring, and healthcare delivery (11), while deep learning techniques have been successfully applied to a wide range of healthcare data sources (12). In addition, conversational AI systems and healthcare chatbots have shown promise for enhancing patient engagement and communication (13). At the same time, recent developments in large language models (LLMs) have created new opportunities for improving emotion recognition in specialized domains. LLMs have demonstrated remarkable capabilities in language understanding, contextual reasoning, and text classification across a wide range of tasks (14). However, deploying LLMs as standalone classifiers may be computationally expensive and impractical for routine implementation, particularly when processing large volumes of clinical dialogue. In contrast, conventional supervised deep learning models offer efficient classification but may struggle with ambiguous or low-confidence predictions, especially when trained on relatively small domain-specific datasets. Recent evidence suggests that reasoning-based language model approaches can improve performance on complex language understanding tasks through more structured contextual analysis (15). A hybrid approach that combines the efficiency of supervised learning with the reasoning capabilities of LLMs may therefore provide an effective solution for emotion recognition in clinical communication contexts.

Therefore, the objectives of this study were twofold. First, we developed an emotion-annotated corpus derived from authentic dentist–patient consultations in restorative dentistry using a systematic annotation framework, expert-guided annotation procedures, and rigorous validation processes. Second, we evaluated a hybrid artificial intelligence framework for automated patient emotion recognition by integrating a supervised deep learning classifier with large language model-based confidence re-ranking. By providing both a domain-specific emotion corpus and an accompanying emotion recognition framework, this study contributes foundational resources for future research in dental informatics, healthcare communication, affective computing, and the development of emotion-aware intelligent systems for dental practice.

## 2 Methodology

### 2.1 Study design overview

**Figure 1:**
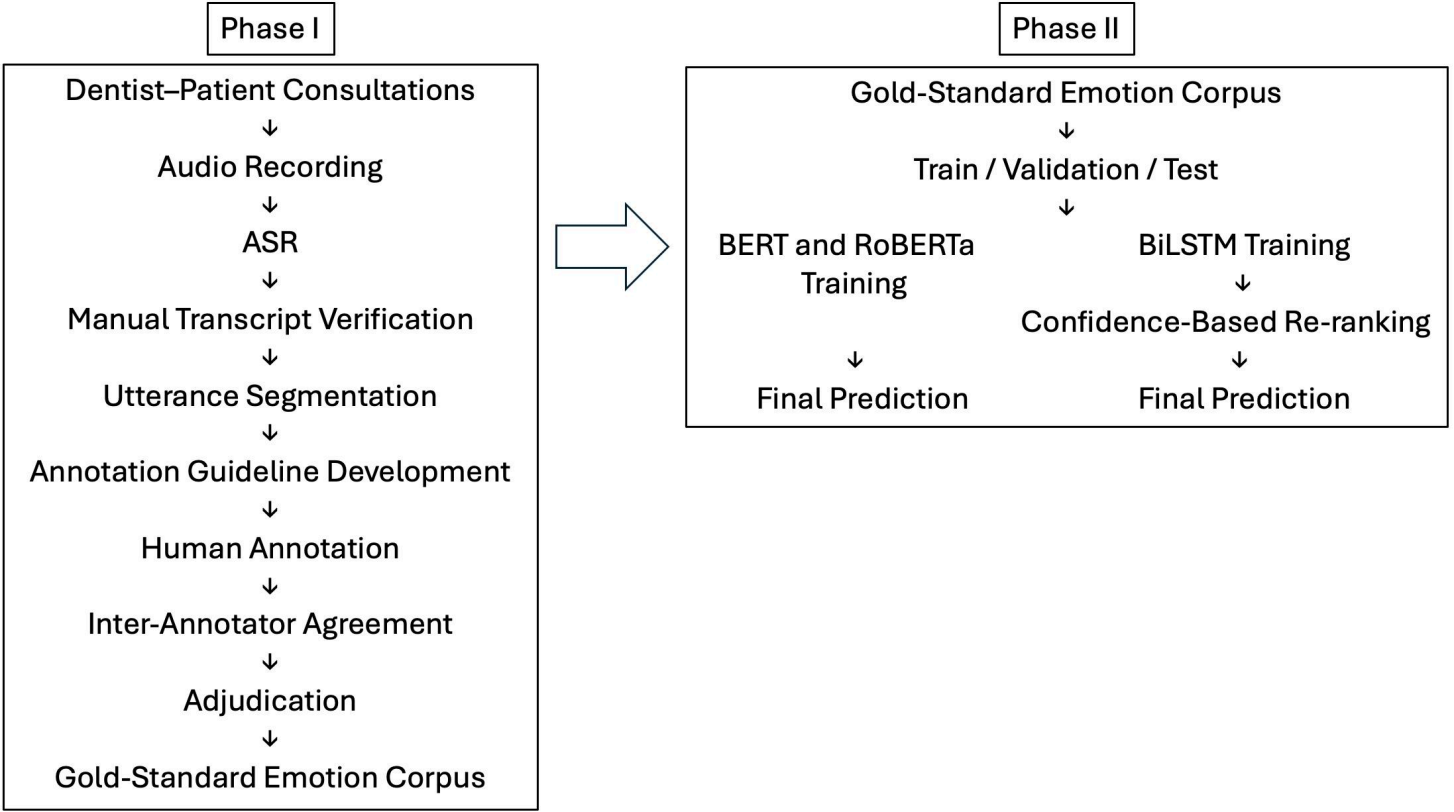
Study design overview.

This study employed a two-phase methodological framework to develop and evaluate an AI system for emotion recognition in dentist–patient consultations. Phase I focused on the development of an emotion-annotated corpus derived from restorative dentistry consultations, including the establishment of annotation guidelines, human annotation procedures, and construction of a gold-standard dataset. Phase II focused on the development and comparative evaluation of multiple artificial intelligence approaches for automated patient emotion recognition. Conventional deep learning and transformer-based baseline models, including BERT and RoBERTa, were first implemented to provide benchmark performance. Building upon these baseline approaches, we developed a hybrid AI framework that combined a supervised deep learning classifier with LLM-based confidence re-ranking to refine prediction confidence and improve emotion recognition. The overall methodological workflow of the study is presented in Figure 1. The process consisted of consultation recording, automatic speech transcription, manual transcript verification, utterance segmentation, emotion annotation, corpus construction, and development of the hybrid emotion recognition framework.

### 2.2 Phase I: emotion-annotated corpus development

#### 2.2.1 Data collection

Ethical approval was obtained from the Trisakti University Faculty of Dentistry institutional review board (No. 1003/S3/KEPK/FKG/9/2025, Date of issue: 19 September 2025). We analyzed data from participants who met the eligibility criteria from 1 October 2025 to 31 January 2026. All procedures were conducted in accordance with ethical standards for research involving human participants. The dentist-patient consultations were collected from restorative dentistry clinics during oral diagnosis and treatment planning sessions. All patients provided written informed consent prior to participation. A total of 12 dentist-patient consultations were collected from restorative dentistry clinics during routine oral diagnosis and treatment-planning sessions. The dataset involved 12 licensed dentists and 12 adult patients. Eligible patients were aged 18 years or older, capable of verbal communication, and able to provide informed consent. Patients with severe cognitive impairment, communication disorders, or incomplete consultation recordings were excluded from the study.

Audio recordings were captured using digital recording devices placed in consultation rooms to ensure high-quality recording of both dentist and patient speech. Audio files were stored in WAV format with a sampling rate of 48 kHz and 24-bit resolution. The average consultation duration was 13.36 min. Only consultations involving diagnosis explanation, clinical examination, and treatment planning were included. Recordings with incomplete audio or significant noise interference were excluded. The corpus development process consisted of five sequential stages: Consultation recording, Automatic speech transcription, Manual transcript verification, Utterance segmentation, Emotion annotation and adjudication. The resulting corpus served as the gold-standard dataset for subsequent machine-learning experiments.

#### 2.2.2 Transcription and data preprocessing

Audio recordings were transcribed using an automatic speech recognition (ASR) system (OpenAI Whisper, large-v3). All transcripts were subsequently manually reviewed and corrected by trained research assistants to ensure transcription accuracy. Transcription errors, omitted words, and speaker-attribution mistakes were corrected manually. A second reviewer performed quality checks on a randomly selected subset of transcripts to ensure consistency. To ensure privacy protection, all transcripts were de-identified. Personally identifiable information, including names, contact details, patient IDs, and other sensitive information, was removed or replaced with anonymized placeholders. Standard text preprocessing was applied, including tokenization and normalization. Non-informative symbols and transcription artifacts were removed when appropriate.

Consultation transcripts were segmented into utterances, defined as the smallest meaningful unit of patient speech expressing a single communicative intent. Segmentation was based on syntactic boundaries, pauses, and conversational turns. Long responses containing multiple emotional expressions were divided into separate utterances when appropriate. For example, the utterance “I am nervous about the procedure, but I am glad it can save the tooth” may be segmented into two separate units corresponding to Fear (“I am nervous about the procedure”) and Happy (“I am glad it can save the tooth”).

Speaker labels were assigned to distinguish between dentist and patient speech. Only patient utterances were retained for emotion annotation and subsequent modeling because the primary objective of the study was to recognize patient emotional states during dental consultations. Dentist utterances were excluded from emotion classification to avoid confounding professional communication behaviors with patient affective responses.

#### 2.2.3 Emotion categories

A six-class emotion taxonomy comprising Angry, Disgust, Fear, Happy, Sad, and Neutral was adopted for corpus annotation. The taxonomy was derived from Ekman's basic emotion framework and adapted to the context of healthcare communication and dental consultations (7). These categories were selected because they encompass both fundamental emotional states and the emotional responses commonly observed during dental diagnosis and treatment discussions. Fear and sadness were particularly relevant because they frequently reflect dental anxiety, uncertainty, and treatment-related concerns. The Neutral category was included to capture informational utterances lacking explicit emotional expression (Table 1).

**Table 1:** Emotion definition.

**Emotion**	**Definition**
Fear	Expression of anxiety, worry, concern, uncertainty
Sad	Expression of disappointment, discouragement
Happy	Positive feelings, relief, satisfaction
Angry	Frustration, irritation
Disgust	Aversion or repulsion
Neutral	Information without clear emotion

#### 2.2.4 Annotation unit and guidelines development

The annotation unit was defined as a single patient utterance. Each utterance was assigned exactly one dominant emotion label. Because patient utterances were generally short and represented a single conversational turn, each utterance was assigned a single dominant emotion label. In cases where an utterance conveyed mixed or ambiguous emotional expressions, annotators discussed the context during the consensus process and assigned the label corresponding to the predominant emotional state expressed by the patient.

Annotation guidelines were developed based on prior work in emotion recognition and clinical communication studies (7–10, 16–18). The guidelines included: Operational definitions of each emotion category, Decision rules for ambiguous cases, Examples of dental-specific emotional expressions, Distinction between symptom reporting and emotional expression, Handling of mixed or subtle emotions. Detailed annotation guidelines and representative examples for each emotion category are provided in the Supplementary File. A pilot annotation study was conducted on 200 utterances to evaluate annotation feasibility and identify ambiguities within the guideline. Annotation disagreements were analyzed and used to refine the operational definitions and decision rules. The guidelines were iteratively refined based on annotator feedback and disagreement analysis.

Three independent annotators with expertise in healthcare communication and linguistic analysis participated in the annotation process. Annotators participated in a structured training program consisting of guideline review, practice annotation sessions, and feedback discussions. Training continued until acceptable agreement levels were achieved on a pilot dataset. Inter-annotator reliability was assessed using Cohen's *κ* coefficient because the annotation process involved three independent annotators assigning mutually exclusive categorical emotion labels to each utterance. According to Landis and Koch, *κ* values between 0.61–0.80 indicate substantial agreement, whereas values above 0.80 indicate almost perfect agreement. Each utterance was independently annotated by three annotators. When two annotators agreed, the majority label was assigned. In cases where all three annotators assigned different labels, the final label was determined through consensus discussion involving all annotators. The adjudicated annotations were used to construct the gold-standard corpus employed for model development and evaluation. All disagreements were reviewed during consensus meetings involving all annotators. Cases that remained unresolved were adjudicated by a senior researcher with expertise in healthcare communication and emotion analysis.

Following adjudication, a gold-standard corpus of patient utterances with validated emotion labels was created. This dataset served as the gold-standard reference corpus for all subsequent machine-learning experiments and performance evaluations. The corpus was divided into training (70%), validation (15%), and test (15%) subsets using stratified sampling to preserve emotion distribution across subsets.

### 2.3 Phase II: development of automated emotion recognition models

Several automated emotion recognition models were developed and evaluated in this study, including transformer-based baseline models and the proposed hybrid framework. The baseline models comprised Bidirectional Encoder Representations from Transformers (BERT) and Robustly Optimized BERT Pretraining Approach (RoBERTa), which were fine-tuned for multiclass emotion classification using the patient–dentist dialogue dataset.

The proposed hybrid emotion recognition framework integrated a supervised deep learning classifier, BiLSTM, with LLM-based confidence re-ranking. The framework was designed to combine the computational efficiency of neural sequence classification with the contextual reasoning capability of LLMs for handling uncertain predictions. The overall architecture consisted of three stages: (1) emotion prediction using a BiLSTM classifier, (2) confidence estimation based on softmax probabilities, and (3) selective LLM-based re-ranking for ambiguous cases with low prediction confidence.

#### 2.3.1 Baseline transformer model

To establish benchmark performance for automated patient emotion recognition, two transformer-based language models, BERT (19) and RoBERTa (20), were implemented as baseline classifiers. Both models were fine-tuned for multiclass emotion classification using the patient–dentist dialogue dataset. Prior to model training, the dialogue transcripts were tokenized using the corresponding pretrained tokenizers, and special classification tokens were added according to each model's input format. The tokenized sequences were then encoded and fed into the transformer encoder, and the contextualized representation of the classification token was passed to a fully connected output layer with a softmax activation function to predict the probability distribution across the emotion classes.

Both BERT and RoBERTa were initialized from their respective pretrained base checkpoints (bert-base-uncased and roberta-base) and fine-tuned using identical hyperparameter settings to ensure a fair comparison. The maximum input sequence length was set to 128 tokens, with a batch size of 16 and a learning rate of 2 × 10⁻⁵. Model parameters were optimized using the AdamW optimizer with a weight decay of 0.01 and a linear learning rate scheduler with a 10% warm-up period. Training was performed for up to five epochs using cross-entropy loss, with early stopping applied if the validation performance did not improve for three consecutive epochs. All experiments were conducted using a fixed random seed of 42 to ensure reproducibility.

#### 2.3.2 Proposed hybrid framework

A BiLSTM network was implemented as the primary emotion classification model (21). A BiLSTM architecture was selected as the baseline classifier because recurrent neural networks remain effective for sequence classification tasks involving relatively small domain-specific datasets. The model also provides computational efficiency and interpretable confidence estimates that can be readily incorporated into the proposed hybrid re-ranking framework.

Patient utterances were transformed into numerical representations prior to model training. Text preprocessing was performed using a Tokenizer to convert each utterance into a sequence of integer word indices. The tokenizer vocabulary was limited to the 10,000 most frequent words, and an out-of-vocabulary (OOV) token was used to represent words not present in the vocabulary. To ensure consistent input dimensions, all sequences were padded or truncated to a fixed length of 50 tokens. Padding was applied to the end of shorter sequences (post-padding), while tokens exceeding the maximum length were removed from the end of longer sequences (post-truncation). The resulting fixed-length sequences were used as inputs to the BiLSTM classifier.

A BiLSTM network was developed to classify patient utterances into six emotion categories. The model consisted of an embedding layer, a bidirectional LSTM layer, dropout regularization, and a dense output layer with softmax activation. The embedding layer followed by a bidirectional LSTM encoder with 128 hidden units in each direction was applied. A dropout layer (dropout rate = 0.5) was applied to reduce overfitting. The encoded representation was passed to a fully connected dense layer and a softmax output layer producing class probabilities for the six emotion categories. All experiments were conducted using a fixed random seed of 42 to ensure reproducibility. The same random seed was used for dataset partitioning, model initialization, and training.

The model was trained using categorical cross-entropy loss and optimized using the Adam optimizer with a learning rate of 0.001. Training was performed using mini-batches of 32 utterances for a maximum of 50 epochs. Early stopping with a patience of five epochs based on validation macro-F1 score was employed to prevent overfitting.

#### 2.3.3 Large language model-based re-ranking strategy

Three open-source instruction-tuned large language models were evaluated: Llama 3.1 8B (22), DeepSeek-R1 8B (23), and Qwen 2.5 7B-Instruct (24). Each LLM independently classified patient utterances into one of the six emotion categories using a standardized prompt:

“Classify the emotion of the following dental patient utterance into one of: Angry, Disgust, Fear, Happy, Sad, Neutral. Output only the label.”

The confidence threshold (*τ*) was optimized using the validation dataset. Candidate threshold values ranging from 0.50 to 0.95 with increments of 0.05 were evaluated. The threshold yielding the highest validation macro-F1 score was selected and subsequently applied to the independent test set. The BiLSTM model first generated emotion predictions along with confidence scores derived from softmax probabilities.

Let:

C_bilstm denote prediction confidence.

*τ* denote confidence threshold.

Decision rules:

If C_bilstm ≥ *τ* → BiLSTM prediction is retained.

If C_bilstm < *τ* → LLM-based re-ranking is triggered.

For low-confidence cases, the selected LLM generated an alternative emotion prediction. The LLM prediction replaced the BiLSTM prediction when the confidence score was below the threshold *τ*.

#### 2.3.4 Experimental design

The corpus was divided using stratified sampling into training (70%), validation (15%), and test (15%) subsets while preserving the distribution of emotion categories. To establish benchmark performance for automated patient emotion recognition, three baseline classifiers were developed and evaluated: Bidirectional Long Short-Term Memory (BiLSTM), Bidirectional Encoder Representations from Transformers (BERT), and Robustly Optimized BERT Pretraining Approach (RoBERTa). The BiLSTM architecture was selected as the backbone of the proposed hybrid framework because of its computational efficiency, suitability for small domain-specific datasets, and compatibility with confidence-based integration strategies.

Four configurations of the proposed hybrid framework were subsequently evaluated to quantify the incremental benefit of LLM-based confidence re-ranking: (1) BiLSTM baseline only, (2) BiLSTM + Llama 3.1 8B re-ranking, (3) BiLSTM + DeepSeek R1 8B re-ranking, and (4) BiLSTM + Qwen 2.5 7B Instruct re-ranking. The performances of these hybrid configurations were compared with those of the standalone baseline models (BiLSTM, BERT, and RoBERTa) using the same training, validation, and test partitions.

All LLMs were evaluated using deterministic inference settings (temperature = 0) to ensure reproducibility. Identical prompts were employed across all LLMs to facilitate fair comparison, and no additional task-specific fine-tuning was performed. The models were implemented using Python and PyTorch, together with the Hugging Face Transformers library for the transformer-based models. Training and inference were conducted on a GPU-enabled workstation equipped with an NVIDIA RTX 4090 graphics processing unit (24 GB VRAM).

#### 2.3.5 Evaluation metrics and statistical analysis

Model performance was evaluated using accuracy, macro-averaged F1-score, weighted F1-score, precision, recall, and class-specific F1-scores. Macro-F1 was designated as the primary evaluation metric because it provides balanced assessment across classes in the presence of class imbalance.

Differences in macro-F1 scores between competing models were evaluated. Bootstrap samples were generated from test-set predictions. Ninety-five percent confidence intervals were calculated for all primary performance metrics. Statistical significance was defined as *p* < 0.05. Confusion matrices were further examined to identify systematic misclassification patterns among emotion categories.

## 3 Results

### 3.1 Emotional corpus characteristics

The final emotion-annotated dental consultation corpus consisted of 2,160 patient utterances derived from 12 restorative dentistry consultations involving 12 dentists and 12 patients. The patient participants ranged in age from 26 to 52 years, with a mean age of 38.3 years. The sample comprised an equal distribution of genders, including six males (50.0%) and six females (50.0%). The participants represented diverse occupational backgrounds, including four freelancers (33.3%), three officers (25.0%), three merchants (25.0%), and two teachers (16.7%). Although the sample included individuals with varied demographic characteristics, all participants were recruited from a single institution and may not fully represent the broader dental patient population.

The total consultation duration was 231 min, with an average utterance length of 7 words. The corpus contained an average of 180 patient utterances per consultation. Following independent annotation and adjudication procedures, inter-annotator agreement achieved a Cohen's *κ* value of 0.89, indicating almost perfect agreement. These annotations were subsequently used to construct the gold-standard dataset for model development and evaluation. Detailed corpus characteristics are presented in Table 2.

**Table 2:** Characteristics of the emotion-annotated dental consultation corpus.

**Characteristic**	**Value**
Number of consultations	12
Number of dentists	12
Number of patients	12
Total duration (minutes)	231
Total patient utterances	2,160
Mean utterance length (words)	7
Mean utterances per Consultation	180

The distribution of emotion categories within the corpus is summarized in Table 3. Among the six emotion categories, Neutral represented the largest proportion of utterances (23.15%), followed by Fear (19.44%) and Disgust (18.98%). In contrast, Happy was the least frequently observed category, accounting for 10.69% of all annotated utterances. The predominance of Neutral, Fear, and Sad utterances suggests that restorative dentistry consultations involve a combination of information exchange and emotionally charged discussions regarding diagnosis, treatment procedures, prognosis, and potential discomfort. The relatively high frequency of Fear-related utterances is consistent with the well-documented prevalence of dental anxiety and concern regarding treatment outcomes.

**Table 3:** Distribution of emotion categories within the dental consultation corpus.

**Emotion**	**Count**	**Percentage**
Fear	420	19.44
Sad	272	12.59
Happy	231	10.69
Angry	327	15.14
Disgust	410	18.98
Neutral	500	23.15

To illustrate the annotation framework, representative examples of patient utterances corresponding to each emotion category are presented in Table 4. These examples demonstrate the diversity of emotional expressions observed during authentic dental consultations and provide insight into the linguistic characteristics associated with each emotion class.

**Table 4:** Representative examples of emotion-annotated patient utterances.

**Emotion**	**Example**
Fear	“I'm worried the procedure will hurt.”
Sad	“I should have come sooner.”
Happy	“I'm glad the tooth can still be saved.”
Angry	“Why wasn't this detected earlier?”
Disgust	“The smell from the infection is unpleasant.”
Neutral	“The pain started last week.”

### 3.2 Performance of baseline and hybrid emotion recognition models

The performance of the baseline BiLSTM classifier and the three hybrid AI configurations is presented in Table 5. Overall, all hybrid models demonstrated improved performance compared with the baseline classifier across multiple evaluation metrics. The baseline BiLSTM model achieved an accuracy of 0.87 and a macro-F1 score of 0.87. Incorporating LLM-based confidence re-ranking increased the macro-F1 score to 0.91, 0.91, and 0.92 for the Llama 3.1, DeepSeek-R1, and Qwen 2.5 hybrid models, respectively.

**Table 5:** Performance comparison of the baseline and hybrid emotion recognition models.

**Model**	**Accuracy**	**Macro-F1**	**Weighted-F1**
BiLSTM	0.87	0.87	0.86
BERT	0.89	0.89	0.88
RoBERTa	0.90	0.91	0.91
BiLSTM + Llama 3.1	0.89	0.90	0.90
BiLSTM + DeepSeek-R1	0.90	0.91	0.91
BiLSTM + Qwen 2.5	0.91	0.92	0.91

Compared with the baseline BiLSTM classifier, the best-performing hybrid model (BiLSTM + Qwen 2.5) improved macro-F1 from 0.87 to 0.92, representing an absolute gain of five percentage points. These findings suggest that confidence-triggered LLM re-ranking can enhance emotion recognition performance by providing additional contextual reasoning for ambiguous utterances.

### 3.3 Class-specific emotion recognition performance

Table 6 presents the class-specific F1-scores for all evaluated models. Overall, the transformer-based and hybrid frameworks consistently achieved higher performance than the baseline BiLSTM model across most emotion categories. The highest classification performance was observed for the Sad and Angry categories, with F1-scores reaching 0.98 across several models, indicating that these emotions were characterized by relatively distinct linguistic expressions in dental consultations.

**Table 6:** Class-specific F1-scores for all models.

**Emotion**	**BiLSTM**	**BERT**	**RoBERTa**	**BiLSTM + Llama 3.1**	**BiLSTM + DeepSeek-R1**	**BiLSTM + Qwen 2.5**
Fear	0.79	0.82	0.84	0.82	0.83	0.85
Sad	0.96	0.97	0.98	0.97	0.98	0.98
Happy	0.92	0.94	0.94	0.94	0.94	0.95
Angry	0.97	0.98	0.98	0.98	0.98	0.98
Disgust	0.84	0.87	0.88	0.88	0.88	0.89
Neutral	0.86	0.88	0.88	0.88	0.88	0.88

In contrast, Fear and Disgust remained the most challenging categories to classify. The baseline BiLSTM model achieved F1-scores of 0.79 and 0.84 for Fear and Disgust, respectively. Performance for these categories improved progressively with the transformer-based and hybrid approaches, with the BiLSTM + Qwen 2.5 framework achieving the highest F1-scores of 0.85 for Fear and 0.89 for Disgust. These findings suggest that the contextual reasoning capabilities of transformer-based and LLM-assisted methods may be particularly beneficial for recognizing emotions that are expressed in subtle, indirect, or ambiguous ways.

For the Neutral category, all advanced models achieved similar performance, with F1-scores of approximately 0.88, indicating that informational utterances without explicit emotional content were comparatively easier to identify. The consistently high performance for Happy, Sad, and Angry, together with the persistent challenges associated with Fear and Disgust, highlights the importance of incorporating contextual information when modeling emotional expressions in dental consultations.

Notably, although Fear was one of the most frequently occurring emotions in the corpus, it consistently exhibited the lowest F1-scores across all models, suggesting that expressions of dental anxiety are often implicit and linguistically similar to other emotional states, particularly Neutral and Disgust.

### 3.4 Effect of confidence-based LLM re-ranking

The impact of the confidence threshold on model performance was assessed. Performance improved as the confidence threshold increased from 0.50 to 0.70, with the macro-F1 score rising from 0.88 to 0.92 (Table 7). The optimal performance was achieved at *τ* = 0.70, where approximately 36% of predictions were redirected to the LLM re-ranking stage. Further increases in the threshold resulted in a gradual decline in performance despite a larger proportion of predictions being routed to the LLM, suggesting that excessive re-ranking may introduce unnecessary label modifications for utterances that had already been correctly classified by the BiLSTM model.

**Table 7:** Performance under different confidence thresholds.

**Confidence Threshold**	**Routed Samples (%)**	**Macro-F1**
0.50	12%	0.88
0.60	22%	0.90
0.70	36%	0.92
0.80	58%	0.91
0.90	82%	0.89

### 3.5 Confusion matrix analysis

**Figure 2:**
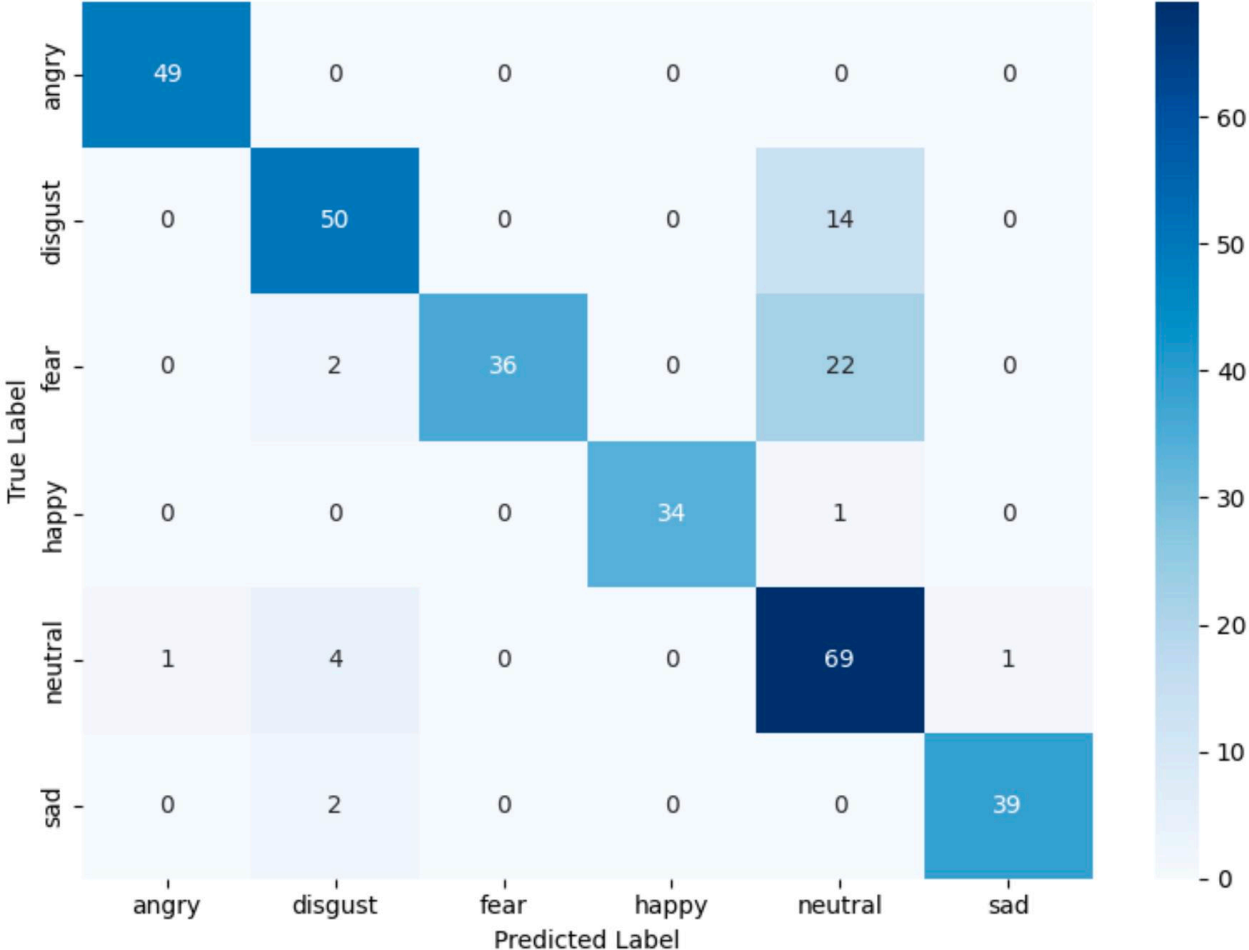
Confusion matrix of the baseline BiLSTM classifier.

**Figure 3:**
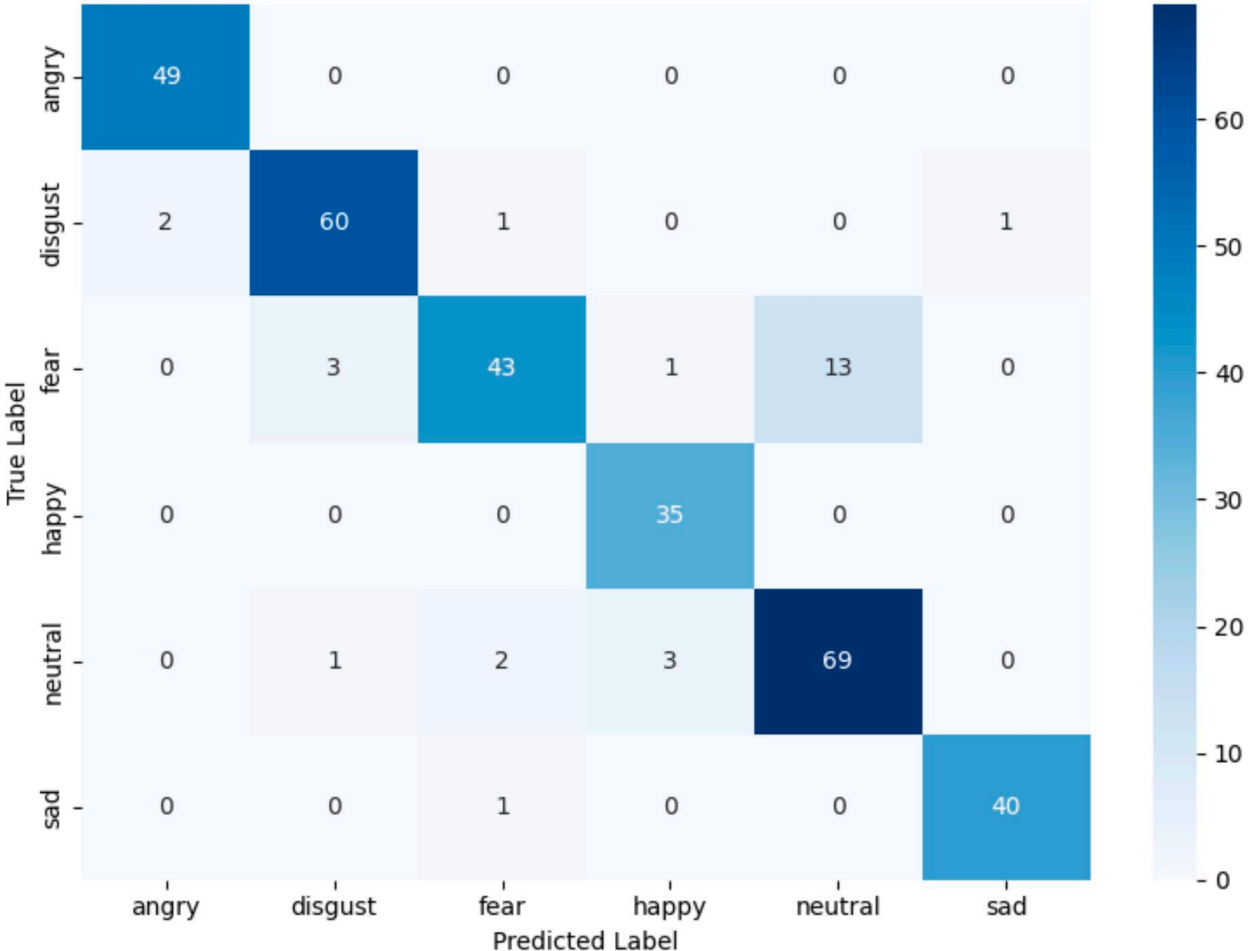
Confusion matrix of the best-performing hybrid emotion recognition model.

Confusion matrices for the baseline BiLSTM classifier and the best-performing hybrid model are shown in Figures 2,3, respectively. The baseline classifier exhibited frequent misclassification among the Fear, Disgust, and Neutral categories, reflecting the semantic similarity and contextual overlap of these emotional expressions in dental consultations. This finding is consistent with the class-specific performance results presented in Table 6, in which Fear and Disgust achieved the lowest F1-scores across all evaluated models.

The hybrid framework reduced several of these confusion patterns, particularly for low-confidence utterances. Misclassifications of Fear and Disgust as Neutral decreased from 36 cases in the baseline model to 13 cases in the hybrid model, suggesting that LLM-based re-ranking provided additional contextual interpretation that was not fully captured by the baseline classifier. Consequently, the F1-score for Fear improved from 0.79 in the BiLSTM model to 0.85 in the BiLSTM + Qwen 2.5 framework, while the F1-score for Disgust increased from 0.84 to 0.89.

Despite these improvements, Fear remained the most difficult emotion to classify. Examination of the confusion matrices revealed that many Fear utterances were still incorrectly classified as Neutral or Disgust. These errors frequently occurred in utterances expressing implicit anxiety or uncertainty, such as concerns about anticipated pain or treatment outcomes, where emotional cues were subtle and context dependent. This observation suggests that recognizing fear-related expressions in dental consultations remains a challenging task and may require richer contextual modeling and larger domain-specific corpora in future studies.

### 3.6 Error analysis

To better understand the limitations of the proposed framework, an error analysis was conducted by examining misclassified utterances produced by the baseline and hybrid models. The majority of errors occurred among the Fear, Disgust, and Neutral categories, which exhibited substantial semantic overlap in the context of dental consultations.

Fear remained the most challenging emotion to recognize despite being one of the most frequently occurring categories in the corpus. Many fear-related utterances were expressed implicitly rather than through explicit emotional statements. For example, patients frequently conveyed anxiety using indirect expressions such as concerns about pain, uncertainty regarding treatment outcomes, or hesitancy toward dental procedures. These utterances often lacked clear lexical indicators of fear and were therefore misclassified as Neutral. Similarly, some utterances expressing aversion toward dental instruments or procedures contained linguistic cues associated with both fear and disgust, resulting in confusion between these two categories.

Representative examples of misclassified utterances are presented in Table 8. Examination of these cases indicated that many classification errors arose from implicit emotional expressions, mixed emotions, and the absence of broader conversational context.

**Table 8:** Representative examples of misclassified utterances.

**True Label**	**Predicted Label**	**Example Utterance**	**Possible Reason**
Fear	Neutral	"Will it hurt a lot?"	Implicit anxiety without explicit emotional words
Fear	Neutral	"I'm not sure if I can tolerate the injection."	Uncertainty expressed indirectly
Fear	Disgust	"I really don't like the sound of the drill."	Simultaneous fear and aversion
Disgust	Neutral	"The smell in the clinic makes me uncomfortable."	Mild emotional expression
Neutral	Fear	"How long will the treatment take?"	Context interpreted as concern

The hybrid framework reduced several of these misclassifications by incorporating contextual reasoning through LLM-based re-ranking. In particular, the framework demonstrated improved recognition of low-confidence utterances that contained subtle emotional cues or required broader contextual understanding. Nevertheless, some errors persisted because certain utterances conveyed mixed emotions or depended on conversational context that was not fully captured within a single utterance.

These findings suggest that patient emotions in dental consultations are often nuanced and context dependent. Future research may benefit from incorporating multi-turn conversational context, multi-label emotion annotation, and larger domain-specific corpora to better model the complexity of emotional expressions in dentist-patient interactions.

### 3.7 Statistical comparison of model performance

Statistical comparisons of model performance were conducted using paired bootstrap resampling (Table 9). Compared with the baseline BiLSTM model, the BERT and RoBERTa models achieved numerically higher macro-F1 scores, with improvements of 0.02 and 0.04, respectively. Similarly, all hybrid frameworks demonstrated numerical performance gains over the baseline, with the BiLSTM + Qwen 2.5 framework showing the largest improvement (Δ macro-F1 = 0.05). However, none of the pairwise comparisons reached statistical significance, as all 95% confidence intervals included zero and all *p*-values exceeded 0.05. Furthermore, no statistically significant differences were observed between the RoBERTa and BiLSTM + Qwen 2.5 models. These findings suggest that although the proposed hybrid framework consistently achieved higher numerical performance, the observed improvements should be interpreted as promising trends rather than definitive evidence of model superiority.

**Table 9:** Statistical comparison of baseline and hybrid models using paired bootstrap resampling.

**Comparison**	***Δ* Macro-F1**	**95% CI**	***p*-value**
BiLSTM vs. BERT	0.02	−0.010 to 0.050	0.09
BiLSTM vs. RoBERTa	0.04	−0.008 to 0.060	0.07
BiLSTM vs. Llama 3.1	0.03	−0.003 to 0.063	0.07
BiLSTM vs. DeepSeek-R1	0.04	−0.002 to 0.082	0.06
BiLSTM vs. Qwen 2.5	0.05	−0.002 to 0.080	0.06
BERT vs. Qwen 2.5	0.03	−0.010 to 0.050	0.07
RoBERTa vs. Qwen 2.5	0.01	−0.015 to 0.035	0.19

### 3.8 Computational cost analysis

To evaluate the practical feasibility of the proposed framework, the computational costs of the baseline, transformer-based, and hybrid models were compared (Table 10). The BiLSTM model exhibited the lowest computational requirements, achieving the fastest inference time owing to its relatively small number of parameters. In contrast, the transformer-based models required substantially greater computational resources, with RoBERTa incurring slightly higher inference latency than BERT due to its larger architecture.

**Table 10:** Computational cost of different models.

**Model**	**Parameters**	**Average inference time (ms/sample)**
BiLSTM	4.1 M	5
BERT	110 M	28
RoBERTa	125 M	31
BiLSTM + Qwen	—	520

The hybrid BiLSTM + Qwen 2.5 framework demonstrated the highest computational cost among all evaluated methods because low-confidence predictions required an additional LLM inference step. Despite this increased computational burden, the selective re-ranking strategy limited the number of utterances processed by the LLM, thereby reducing the computational overhead compared with routing all samples to the language model. These findings indicate that the proposed framework achieves a favorable trade-off between predictive performance and computational efficiency, as it leverages the contextual reasoning capabilities of large language models only when the confidence of the primary classifier is low.

## 4 Discussion

This study developed an emotion-annotated dental consultation corpus derived from authentic restorative dentistry consultations and evaluated a hybrid AI framework for automated patient emotion recognition. The resulting corpus comprised 2,160 patient utterances spanning six emotion categories and demonstrated high annotation reliability (*κ* = 0.89). Furthermore, integrating confidence-triggered LLM re-ranking with a BiLSTM classifier improved macro-F1 performance from 0.87 to 0.92, suggesting that selective use of large language models can enhance recognition of emotionally ambiguous utterances.

A major contribution of this study is the development of a domain-specific emotion corpus derived from authentic dentist–patient consultations. Existing emotion recognition research has largely relied on datasets collected from social media platforms, movie dialogues, customer service interactions, or general conversational settings (8–10). Although several healthcare-related corpora have been reported, few have focused specifically on dental communication (16–18). Dental consultations involve unique emotional contexts, including anxiety regarding pain, uncertainty about treatment outcomes, concerns about treatment costs, and relief following diagnosis or treatment planning. By capturing these domain-specific emotional expressions, the present corpus provides a valuable resource for future research in dental informatics, healthcare communication, and affective computing.

The distribution of emotions observed in the corpus provides insight into the emotional landscape of restorative dentistry consultations. Neutral utterances were the most common category, reflecting the substantial amount of information exchange that occurs during diagnosis and treatment planning. However, Fear emerged as the second most prevalent emotion, accounting for nearly one-fifth of all patient utterances. This finding is consistent with the extensive literature on dental anxiety, which identifies fear of pain, invasive procedures, and uncertainty regarding treatment outcomes as major barriers to dental care (2, 3). The substantial proportion of fear-related utterances underscores the importance of emotional awareness and communication skills in dental practice.

An important finding was that Fear was both one of the most frequently occurring emotions and the most challenging category to classify. Although Fear accounted for 19.44% of all utterances, it achieved the lowest F1-score among the six emotion categories. This observation suggests that expressions of dental anxiety are linguistically heterogeneous and often overlap with other negative emotional states such as concern, uncertainty, frustration, or disgust. Unlike emotions such as sadness or anger, which may be communicated through more explicit linguistic cues, fear-related expressions are often subtle and context-dependent (10). The difficulty in recognizing Fear highlights an important challenge for both human clinicians and automated emotion recognition systems and suggests that future models may benefit from incorporating broader conversational context and multimodal information.

The proposed hybrid framework consistently achieved higher performance than the baseline BiLSTM classifier across all evaluation metrics, although the pairwise improvements did not reach the predefined threshold for statistical significance. The best-performing configuration, which combined BiLSTM classification with Qwen 2.5-based confidence re-ranking, achieved a five-percentage-point improvement in macro-F1 score. These findings support the hypothesis that confidence-triggered LLM intervention can provide complementary contextual reasoning when conventional neural classifiers encounter ambiguous utterances. This observation is broadly consistent with the affective computing literature, which emphasizes that accurate emotion recognition depends on contextual interpretation rather than isolated lexical cues (4). Furthermore, recent studies have demonstrated that large language models possess remarkable language understanding capabilities and can perform classification tasks through contextual inference and reasoning (14). The ability of LLMs to analyze nuanced emotional expressions may explain the improved performance observed for low-confidence utterances in the present study. Importantly, the hybrid framework did not replace the baseline classifier but selectively invoked LLM reasoning only for low-confidence predictions. This strategy offers a practical balance between computational efficiency and classification accuracy and may be particularly advantageous for domain-specific datasets where training data are relatively limited. The effectiveness of this approach may also be related to the emergent reasoning capabilities reported in modern LLMs, whereby stepwise contextual analysis can improve decision-making in ambiguous language understanding tasks (15).

The effectiveness of the proposed framework appears to stem from the complementary strengths of the two components. The BiLSTM classifier efficiently captures common lexical and sequential patterns in patient utterances, whereas the LLM provides additional contextual reasoning for low-confidence predictions that involve subtle, implicit, or ambiguous emotional expressions. By selectively invoking the LLM only for uncertain cases, the framework preserves computational efficiency while leveraging the broader semantic knowledge of large language models. The threshold analysis further demonstrated that excessive re-ranking may degrade performance by unnecessarily modifying predictions that were already correctly classified, highlighting the importance of confidence-based selective routing.

Beyond automated emotion recognition, the developed corpus and hybrid framework may have applications in dental education and communication training. Emotion-aware AI systems could assist educators in evaluating students' communication skills, identifying missed emotional cues, and providing personalized feedback during simulated patient interactions. In clinical settings, automated recognition of patient emotions may support communication analysis, facilitate patient-centered care, and contribute to the development of intelligent decision-support systems capable of identifying patients experiencing anxiety or emotional distress during consultations.

Several limitations should be acknowledged. First, the corpus was derived from a relatively small number of consultations conducted within a single clinical context, which may limit generalizability to other dental specialties and patient populations. Although the study included participants with diverse ages, balanced gender representation, and varied occupational backgrounds, all consultations were obtained from a single institution and may not fully capture the linguistic, cultural, and socioeconomic diversity of the broader dental patient population. Consequently, the emotional expressions represented in the corpus may not be entirely representative of those encountered in different clinical settings or geographic regions. Future studies should validate the proposed framework using larger, multi-center datasets encompassing more heterogeneous patient populations and a wider range of dental specialties to improve the robustness and external validity of the findings. Second, emotion annotation was based solely on textual transcripts and did not incorporate vocal, facial, or physiological cues that may convey additional emotional information. Another methodological consideration relates to the use of a single-label emotion taxonomy. Although emotional experiences in clinical interactions are often complex and may involve co-occurring emotions, the present study assigned one dominant emotion label to each utterance because patient responses were typically brief and represented a single conversational turn. This approach simplified the annotation process and facilitated supervised classification; however, it may not fully capture the multidimensional nature of human emotions. Some utterances may simultaneously express, for example, fear and sadness or fear and disgust, resulting in the loss of nuanced emotional information. Future studies may benefit from exploring multi-label emotion annotation frameworks or dimensional emotion representations to better characterize the complexity of emotional communication in dental consultations. Third, although the hybrid framework demonstrated consistent performance improvements, statistical significance was not achieved, likely due to the limited size of the evaluation dataset. Future studies should expand the corpus across multiple institutions and dental disciplines, incorporate multimodal emotional signals, and evaluate transformer-based architectures and larger language models to further improve emotion recognition performance.

## 5 Conclusion

This study developed a novel emotion-annotated dental consultation corpus derived from authentic restorative dentistry consultations and evaluated a hybrid artificial intelligence framework for automated patient emotion recognition. The resulting corpus comprised 2,160 patient utterances spanning six emotion categories and demonstrated high annotation reliability, providing a valuable domain-specific resource for future research in dental communication and affective computing. The findings revealed that Fear was both a prevalent and challenging emotion to recognize, highlighting the complexity of detecting dental anxiety from patient language. Although the proposed confidence-triggered hybrid framework, which selectively combined BiLSTM classification with large language model-based re-ranking, achieved the highest numerical performance among the evaluated models, the observed improvements were not statistically significant. Nevertheless, the results suggest that integrating efficient neural classifiers with targeted LLM reasoning represents a promising strategy for recognizing emotionally ambiguous utterances and may provide complementary contextual information beyond conventional supervised approaches. Collectively, the developed corpus and hybrid framework establish a foundation for future research on emotion-aware applications in dental education, communication training, patient-centered care, and intelligent clinical support systems. Further studies involving larger, multi-center, and more diverse datasets are warranted to validate the generalizability and practical utility of the proposed approach.

## Data Availability

The raw data supporting the conclusions of this article will be made available by the authors, without undue reservation.
