# Supplementary material for: Hybrid artificial intelligence framework for patient emotion recognition in dentistry
Source: Front Oral Health. 2026 Jul 29;7:1909407. doi: 10.3389/froh.2026.1909407 (PMC13461485; doi:10.3389/froh.2026.1909407)
Supplement: Supplementary file 1 [file Datasheet1.pdf]

Annotation Guidelines for Emotion Labelling of Patient Utterances in Dental Consultations

1. Purpose

These guidelines describe how to annotate each **patient utterance** with one of six emotion categories: Neutral, Fear, Sad, Happy, Angry, Disgust

The goal is to identify the **primary emotion explicitly or implicitly expressed in the patient's utterance**, rather than diagnosing the patient's psychological state. Annotation should rely on the linguistic content of the utterance and, where available, conversational context.

Each utterance should receive **only one emotion label**.

2. General Annotation Principles

2.1 Annotate the expressed emotion, not the situation

Label the emotion that the patient expresses, rather than the emotion that seems reasonable given the situation.

Example

Patient:

"I have to get a tooth extracted today."

Label:

Neutral

Rationale:

The situation may be frightening, but no emotion is expressed.

2.2 Use explicit evidence whenever possible

Prefer labels supported by clear lexical, grammatical, or discourse cues.

Examples of emotional markers include: emotion words, evaluative language, exclamations, intensifiers, hesitation caused by anxiety, emotional descriptions, emotional reactions to treatment. Avoid inferring emotions solely from the clinical context.

2.3 One label per utterance

Assign the **dominant emotion** expressed.

If multiple emotions appear, identify the one that is most prominent according to the decision rules in Section 5.

3. Emotion Categories

3.1 Neutral

Operational Definition

The utterance contains **no clear emotional expression**. It primarily communicates information, symptoms, medical history, preferences, or asks questions without conveying an emotional attitude.

Neutral does **not** mean the patient feels no emotion; rather, no emotion is linguistically expressed.

Typical Characteristics

- reports symptoms
- answers clinician's questions
- asks factual questions
- gives medical history

- discusses logistics
- describes procedures objectively

Examples
"My tooth has been hurting for three days."
"The pain is on the left side."
"I brushed twice this morning."
"How long will the filling take?"
"I can't open my mouth very wide."

3.2 Fear

Operational Definition
The patient expresses anxiety, nervousness, worry, apprehension, dread, or concern regarding dental treatment, pain, injections, diagnosis, or future outcomes.
Fear may be explicit or indirectly expressed through uncertainty and avoidance.

Typical Linguistic Indicators
- scared
- afraid
- nervous
- worried
- anxious
- terrified
- panic

Examples
"I'm really scared of the injection."
"I'm worried the extraction will be painful."
"Can you stop if it hurts?"
"I don't think I can do this."
"I'm shaking just thinking about it."

3.3 Sad

Operational Definition
The patient expresses disappointment, hopelessness, discouragement, emotional distress, regret, or sorrow related to their dental condition or treatment.
Sadness concerns emotional suffering rather than physical pain.

Typical Linguistic Indicators
- upset
- depressed
- miserable
- disappointed
- hopeless
- embarrassed

Examples
"I'm so upset that I lost another tooth."
"I hate smiling because of my teeth."
"I wish I had taken better care of them."
"It's really depressing."

"I'm embarrassed to talk to people."

3.4 Happy

Operational Definition

The patient expresses positive emotion including happiness, relief, satisfaction, gratitude, excitement, or confidence.

Relief after treatment is included within this category.

Typical Linguistic Indicators

- happy
- relieved
- glad
- excited
- thankful
- appreciate
- wonderful
- much better

Examples

"I'm so relieved it's over."
"Thank you, I feel much better."
"I'm happy with how my teeth look."
"That didn't hurt at all."
"I'm excited to get my new smile."

3.5 Angry

Operational Definition

The patient expresses irritation, frustration, blame, resentment, or anger toward the dentist, clinic, previous treatment, waiting time, cost, or circumstances.

Typical Linguistic Indicators

- angry
- annoyed
- frustrated
- unacceptable
- ridiculous
- unfair
- blame
- complaints with emotional tone

Examples

"I've been waiting for over an hour."
"Nobody explained this to me."
"I'm really frustrated with this treatment."
"This shouldn't have happened."
"The last dentist ruined my tooth."

3.6 Disgust

Operational Definition

The patient expresses revulsion, aversion, nausea, or strong dislike toward dental sensations, smells, tastes, blood, pus, or oral conditions.
Disgust differs from fear because the dominant response is revulsion rather than anticipation of harm.

Typical Linguistic Indicators

- disgusting
- gross
- nasty
- revolting
- makes me sick
- can't stand the taste
- gagging

Examples

"The taste is disgusting."
"The smell makes me feel sick."
"I almost gag whenever you use that material."
"There's a horrible taste in my mouth."
"Seeing the blood makes me nauseous."

4. Distinguishing Symptom Reporting from Emotional Expression

One of the most common annotation challenges is distinguishing physical symptoms from emotional states.

Label as Neutral when the patient only reports symptoms.

Examples
"My tooth hurts."
Neutral
"My gums are swollen."
Neutral
"It hurts when I chew."
Neutral
Pain alone is **not** an emotion.

Label as Emotional when the patient evaluates or reacts emotionally to symptoms.

Examples
"I'm scared because the pain keeps getting worse."
Fear
"I'm really upset that I can't eat properly."
Sad
"This pain is driving me crazy."
Angry
"The smell from the infection makes me sick."
Disgust

5. Decision Rules for Ambiguous Cases

Rule 1. Prioritize explicitly expressed emotion.

Explicit emotional words outweigh inferred emotions.

Example
"The extraction is tomorrow."
Neutral
not Fear

Rule 2. Questions are not automatically Fear.
Example
"How long will the procedure take?"
Neutral
Example
"Will it hurt?"
Fear
because concern about anticipated pain is expressed.

Rule 3. Physical pain alone is Neutral.
Example
"My tooth hurts."
Neutral
Example
"I'm terrified because it hurts so much."
Fear

Rule 4. Relief belongs to Happy.
Example
"I'm so relieved it's finally over."
Happy

Rule 5. Frustration belongs to Angry.
Example
"This is so frustrating."
Angry
even if the patient is not shouting or insulting anyone.

Rule 6. Embarrassment usually belongs to Sad.
Example
"I'm embarrassed to smile."
Sad
unless another emotion clearly dominates.

Rule 7. Revulsion belongs to Disgust.
Example
"The pus tastes disgusting."
Disgust
not Fear.

6. Handling Mixed or Subtle Emotions

Some utterances contain more than one emotional cue.
Annotate the **dominant** emotion.
Example
"I'm scared, but I'm glad I finally came."
Primary emotion:
Fear
because anxiety drives the utterance.

Example
"I'm still angry, although I'm relieved it's fixed."
Primary emotion:
Angry
because the complaint remains central.

Example
"I guess it'll be okay."
This may indicate mild reassurance rather than clear happiness.
Recommended label:
Neutral
unless additional context indicates emotional relief.

Example
"Hopefully it won't hurt."
Fear
The statement reflects anticipatory concern.

7. Contextual Annotation
Annotators may consider previous conversational turns when the current utterance is ambiguous.
Example
Dentist:
"Are you nervous?"
Patient:
"Very."
Label:
Fear
The emotion is only interpretable using context.
However, context should **clarify** the utterance rather than override its linguistic content.
